# Aqueous zinc batteries: Design principles toward organic cathodes for grid applications

**DOI:** 10.1016/j.isci.2022.104204

**Published:** 2022-04-04

**Authors:** Eloi Grignon, Alicia M. Battaglia, Tyler B. Schon, Dwight S. Seferos

**Affiliations:** 1 Department of Chemistry, University of Toronto, 80 St. George Street, Toronto, ON M5S 3H6, Canada; 2 Department of Chemical Engineering and Applied Chemistry, University of Toronto, 200 College Street, Toronto, ON M5S 3E5, Canada; 3 e-Zn Inc., 25 Advance Road, Toronto, ON M8Z 2S6, Canada

**Keywords:** Electrochemistry, Energy systems, Energy manageme

## Abstract

The development of low-cost and sustainable grid energy storage is urgently needed to accommodate the growing proportion of intermittent renewables in the global energy mix. Aqueous zinc-ion batteries are promising candidates to provide grid storage due to their inherent safety, scalability, and economic viability. Organic cathode materials are especially advantageous for use in zinc-ion batteries as they can be synthesized using scalable processes from inexpensive starting materials and have potential for biodegradation at their end of life. Strategies for designing organic cathode materials for rechargeable zinc-ion batteries targeting grid applications will be discussed in detail. Specifically, we emphasize the importance of cost analysis, synthetic simplicity, end-of-life scenarios, areal loading of active material, and long-term stability to materials design. We highlight the strengths and challenges of present zinc-organic research in the context of our design principles, and provide opportunities and considerations for future electrode design.

## Graphical abstract

**Figure:**
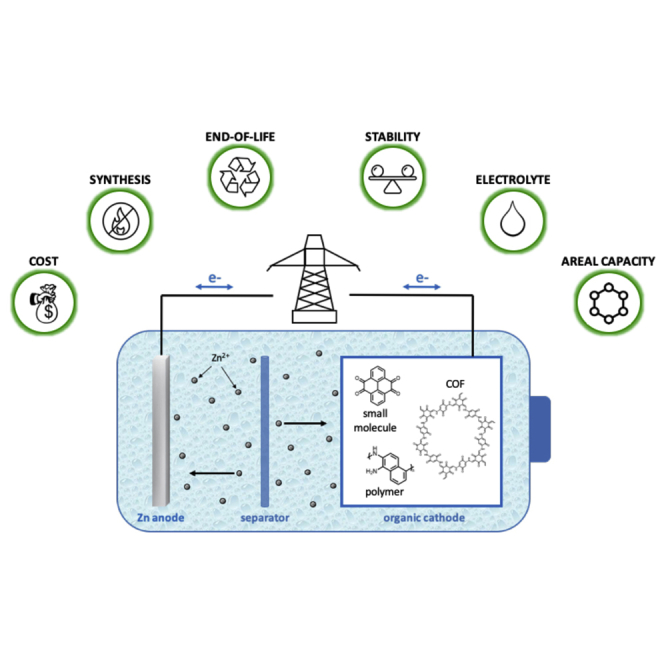

## Introduction

The development of a sustainable electrical grid capable of fueling electric vehicles, homes, and industry is crucial for reducing anthropogenic greenhouse gas emissions. In the past decades, technological developments and economies of scale have sufficiently lowered the cost of renewable electricity to achieve grid parity with fossil fuels in many parts of the world, thereby providing the required input of clean electricity (Poizot et al., 2020). However, due to the intermittency of renewable sources of energy, such as solar and wind, a global transition to these sources is contingent upon the availability of a reliable, low-cost energy storage technology.

Currently, 98% of global grid energy storage is captured in the form of pumped hydroelectric power, which relies on the gravitational potential energy stored between two reservoirs of water at different altitudes (Poizot et al., 2020). Owing to the strict geographical and spatial requirements of pumped hydroelectric as well as its modest efficiency, intensive research is targeted at developing alternative energy storage systems that are more compact and more performant. Specifically, batteries are promising candidates due to their modular nature and potential for scaling up to the capacities that will be required in coming decades (Dunn et al., 2011).

Today’s battery market is dominated by lithium-ion batteries (LIBs) due to their exceptionally high energy density and long cycle life. Although energy and power density are key factors for batteries targeting transportation, they are relatively less important for grid applications. Instead, the cost, safety, and sustainability of the technology are far more significant due to the scale of storage required and the relaxed constraints on volumetric energy density. In this regard, lithium-ion batteries do not fare well. The scarcity of lithium translates to high costs, while the inherent safety concerns posed by flammable organic electrolytes present a serious barrier to adoption for large-scale grid storage. Compounded with this are geopolitical issues pertaining to the uneven global distribution of lithium deposits, which could lead to severe shortages in certain markets if demand rises. Finally, with the increasing prevalence of electric vehicles, lithium-ion battery supply will be mostly allocated for this use, thereby leaving a void in the energy storage market for grid applications.

Zinc-ion batteries (ZIB) offer an exciting alternative due to the use of metallic zinc anodes, which have a high volumetric capacity (5855 mAh cm^−3^) and gravimetric capacity (820 mAh g^−1^), a higher natural abundance (75 ppm in the Earth’s crust vs. 20 ppm for lithium), low cost, and inherent safety due to the lack of both toxic materials and flammable solvents (Zhang et al., 2020b). Although the higher redox potential of Zn relative to Li (−0.76 V and −3.04 V vs SHE, respectively) entails a loss in energy density, it also endows ZIBs with certain practical advantages that render them particularly conducive to large-scale manufacturing. For instance, Zn is considerably more stable in ambient conditions, which means that fabrication processes need not be hindered by the constraints and energy requirements of moisture-free environments. More importantly, the high redox potential of Zn enables its compatibility with aqueous electrolytes, which not only have very high ionic conductivities but also lower the cost of the system and provide intrinsic safety benefits over the conventional organic solvents used in LIBs. The use of aqueous electrolytes eliminates the risk of fires or explosions from flammable components, which is critical for grid storage systems placed in densely populated areas. Finally, a recent life cycle assessment showed that the global warming potential of aqueous ZIBs can be up to 80% lower than that of LIBs, which suggests that they are a much more sustainable alternative (Iturrondobeitia et al., 2022). Therefore, despite their lower energy density, aqueous ZIBs operating near neutral pH values are well suited for applications where safety, cost, and carbon footprint are the critical factors (Blanc et al., 2020).

The ideal battery system for grid storage should have a long cycle life, low cost, and components that have dependable supply chains. Recently, the ambitious target to bring grid storage costs below $100/kWh has been suggested for technologies to be competitive (Gür, 2018). In accordance with the visionary article proposed by Larcher and Tarascon (2015) on the need for a paradigm shift in the design of batteries, we believe that a change from conventional, high-performance materials to low-cost, sustainable electrodes is necessary to fully exploit the potential advantages of ZIBs for grid storage applications. In this respect, we envision organic materials occupying a central role in the future of grid-scale energy storage through their use as aqueous ZIB cathodes. In the following sections, we address the benefits of utilizing organic materials in ZIBs, highlight the design principles that must be considered for these batteries to achieve widespread grid application, and showcase several promising results that have been achieved with organic cathode materials.

## Discussion

Research on secondary aqueous ZIBs traces its origins to a seminal report from Kang and coworkers, where reversible Zn-ion intercalation was shown to occur in ⍺-MnO_2_ (Xu et al., 2012). Key to this work is the use of mild electrolytes, which enables the anodic plating of Zn-ions in favourable (i.e not dendritic) morphologies. Since then, a host of other inorganic materials have been proposed as ZIB cathodes. Briefly, these can be categorized into several families, such as V-rich oxides (Kundu et al., 2016; Yan et al., 2018; Zhang et al., 2018; Zhou et al., 2018), Mn-rich oxides (Pan et al., 2016; Zhang et al., 2017; Chao et al., 2019), Prussian blue analogs (Trócoli and La Mantia, 2015; Ma et al., 2019), and spinel phases (Zhang et al., 2016; Pan et al., 2018).

Despite the highly promising performance of many inorganic cathode materials, they have intrinsic limitations that may prove problematic for their implementation in the next generation of sustainable batteries. For instance, the use of harmful and toxic elements as well as the energy-intensive syntheses required to make these cathodes directly mitigate the environmental gains that ZIBs are designed to achieve. From the perspective of performance, the intercalation charge storage mechanism of inorganic cathodes can also be problematic. This is largely due to the higher charge of Zn ions, which precludes efficient solid-state diffusion, thereby leading to sluggish kinetics during routine operation of many inorganic-based ZIBs (Canepa et al., 2017; Poizot et al., 2020; Zhang et al., 2020b). Finally, large volume changes and active material dissolution in inorganic cathodes can often lead to poor cycling stability.

In light of these limitations, we consider organic cathodes to be ideal materials for use in aqueous ZIBs. These materials can be sourced from inexpensive, abundant feedstocks such as biomass, and can be synthesized via low-temperature solution-based processes (Schon et al., 2016; Zhao et al., 2018). All of these factors work in tandem to lower the system’s cost, which may also encapsulate the environmental and safety costs associated with the technology. A low cost per kWh stored should be the main priority when designing materials targeting grid applications. Currently, organic cathodes are already cost-competitive with inorganic materials commonly used in ZIBs. For example, pyrene-4,5,9,10-tetraone and poly(anthraquinonyl sulfide), both of which are viable ZIB cathodes (Dawut et al., 2018; Guo et al., 2018), are estimated to cost $4–6/kg and $3–4/kg at a 500 kg scale, respectively (Liang et al., 2017). Although factors such as energy density and the need for conductive additives should be considered, these prices are comparable to the widely employed MnO_2_ ($1.7/kg) and V_2_O_5_ ($5.5/kg) (Zhang et al., 2020b).

When assessing the cost or environmental footprint of a material, it is important to consider its impact over its entire lifetime, as is often done in life cycle assessments. In this regard, the possibility to have recyclable or biodegradable functionalities within the organic cathode structure entails great potential for low-cost and sustainable end-of-life scenarios for these materials.

**Figure 1:**
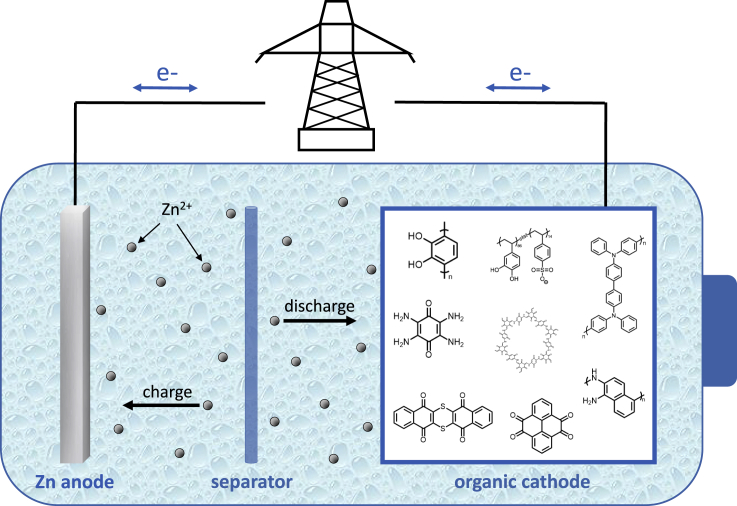
Schematic of a zinc-ion battery During discharge, the organic cathode is reduced with associated uptake of Zn^2+^. During charge, the cathode is oxidized and Zn^2+^ returns to the Zn anode, where they are plated.

Finally, organic materials typically store charge through coordination reactions rather than the insertion of multivalent cations (Figure 1). These reactions typically display much faster kinetics than intercalation, and therefore allow for the construction of high-power devices. Bypassing solid-state insertion also means that there are no cathodic volume changes associated with the battery’s discharge, which allows for much higher stabilities.

### Design principles

**Figure 2:**
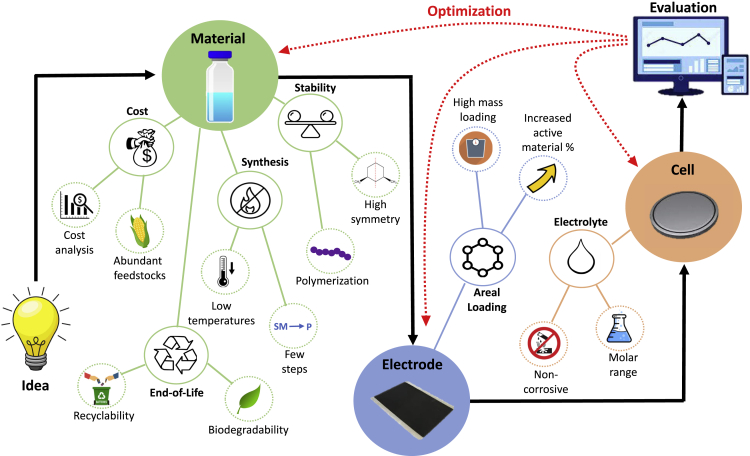
Design process and strategies to consider when developing organic ZIB cathodes Design occurs on three levels: material synthesis, electrode assembly, and cell fabrication. This process is iterative and so researchers may be required to optimize their material, electrode, or cell after evaluation.

Many groups have already exploited the advantages of organic materials to design aqueous ZIBs with organic cathodes (Cui et al., 2020). However, reporting conventions tend to follow those of LIBs, where gravimetric capacity and high rate capability are showcased, even if these metrics are not the most pertinent for grid applications. Below, we propose a set of design principles to guide researchers when creating aqueous ZIBs for grid storage (Figure 2).

### Cost: $/kWh as a fundamental metric

The cost per unit of stored energy is the key criterion for the adoption of a technology for grid applications. Although aqueous ZIBs are inherently less expensive than LIBs, lowering the price will remain critical if they are to be adopted over competing technologies such as redox flow, sodium-ion, or liquid-metal batteries. As such, we propose that it is critical to assess the cost of the input materials relative to the energy stored over the device lifetime when designing new organic cathode materials for ZIBs. This evaluation may either take the form of a life cycle cost analysis, or may be a quick, approximative assessment used to supplement a scientific publication, as was recently demonstrated by Molina et al. (2020).

Of course, the cost per kWh ratio can also be lowered by increasing the energy density. As such, the voltage and capacity of electrode materials remain important metrics, while the cycling rate and number of cycles contextualize these numbers. Many batteries employ auxiliary elements such as carbon supports (e.g. graphene, carbon nanotubes) or Nafion membranes in order to boost the long-term energy density. This is typically achieved by increasing conductivity or limiting dissolution, which allows the device to retain capacity at high current densities and cycle numbers, respectively (Zhao et al., 2018). In these situations, it is especially important for researchers to evaluate whether the performance gains achieved by adding these expensive components to the system justify their added price. We expect that in certain cases, the additional cost presents a barrier to application rather than a solution.

### Simplicity, scalability, and safety of synthesis

It is critical to consider the various facets of a material’s synthesis, and how well they translate to an industrial process. For instance, syntheses should be limited in steps in order to minimize associated costs with labor, reagents, solvents, and the disposal of byproducts. Moreover, syntheses that avoid the use of expensive catalysts or high temperatures are conducive to the scales required for grid application. In this respect, the principles of green chemistry have brought forth a tremendous catalog of low-impact reactions enabling the production of functional materials (Li and Trost 2008). Finally, the ability to develop production from the laboratory scale to the industrial scale should be considered. Here, factors such as concentration, reaction viscosity, time, exothermicity, and the need for inert atmospheres are of great importance.

Many practical syntheses for organic cathodes have already been reported. In one case, Wang and coworkers obtained the quinone DTT through the gram-scale one-pot condensation of inexpensive 2,3-dichloro-1,4-naphthoquinone and sodium sulfide in water at 60°C (Wang et al., 2020b). The mild reaction conditions, the absence of intense purifications, and the tolerance to ambient environment make this preparation highly scalable, although it should be noted that no yield was reported. In another example, Liu and coworkers electropolymerized their active material, 1,5-naphthalenediamine, directly onto the carbon fabric current collector (Zhao et al., 2020). Here, low-cost electricity is used as the driving force of the reaction while minimizing the required steps and materials. Interestingly, some commercially available materials can directly be used as ZIB cathodes without further molecular modification, as was demonstrated by Kundu et al. with *p*-chloranil ($220 per kg from TCI) (Kundu et al., 2018). Although the authors eventually had to confine the chloranil cathode into mesoporous CMK-3 to avoid a phase evolution, this work illustrates that performant cathodes do not necessarily require complicated or expensive syntheses.

### End-of-life scenarios

Although seldom reported upon, the end-of-life scenario of battery materials is key in determining the potential gains achievable by an energy storage technology. For instance, the lack of a recycling industry has limited the environmental benefits of electric vehicle LIBs—most of these batteries end up in landfill, which represents a tremendous waste of material as well as a serious hazard to human health and the environment. In practice, the development of a recycling industry is shaped by its economic outlook, which is dependent on the cost of the process (transport of batteries, mechanical/chemical separation of components, and materials recovery) and on the value of recovered products (Harper et al., 2019).

At first glance, recycling organic materials from aqueous ZIBs has attractive features; the intrinsic safety of the devices should lower the process cost and complexity while clever molecular design can promote on-demand degradation, as has recently been demonstrated for a polypeptide-based battery (Nguyen et al., 2021). However, as organic electrodes are inherently inexpensive relative to cathodes containing high-value transition metals such as nickel or cobalt, the financial motivation for recycling is largely mitigated. As a result, the chemical recycling of electrodes is likely to occur only if incentivized externally (e.g. through government programs) or as a result of technological breakthroughs. Therefore, we view the design of biodegradable materials as a more realistic path toward sustainable organic cathodes. In such a scenario, the end-of-life benefit is two-fold; the molecular weight reduction of the electrode materials limits their persistence in the environment while the methane produced from degradation can potentially be captured and utilized, thus adding an energy credit to the overall life cycle of the material (Powell et al., 2016).

Standard protocols have been developed to test and benchmark material biodegradation. Typically, the volume of gas generated is measured and used to calculate the percentage of biodegradation that has occurred in the sample. It is important to conduct these biodegradation tests in a realistic environment. For example, electrode materials that will likely end up in landfill should be studied under anaerobic conditions to best capture the available degradation pathways that the material will face at end of life. Standard testing protocols for various settings are available, such as the ASTM D5526-18 for landfills and the ASTM D6400 for municipal composting (Kolstad et al., 2012).

### Areal loading

It is typical in both industrial and research settings to mix the active electrode material with a conductive carbon additive. In commercial cells, this additive generally makes up about 2% of the total cathode mass. However, due to their low electronic conductivity, organic materials generally require much larger proportions of additive in the cathode in order to maintain tolerable performances. In our laboratory, we have used cathode formulations employing 30%–60% of additive for lithium-ion batteries (An et al. 2020, 2022; McAllister et al., 2021), and it is not uncommon to see formulations with up to 70% of additive in the literature. Because specific capacities are reported per mass of active material rather than total electrode mass, this reduction in effective capacity is sometimes overlooked. Therefore, increasing the proportion of active material in the cathode composite represents a viable route toward increasing energy density, and should be a key area to target for future research.

Another key parameter is the mass loading per unit area of the electrode. In lab settings, the loading is typically below 2 mg cm^−2^, which allows researchers to extract the best possible performance from a given material. However, commercial cells usually employ mass loadings of 10 mg cm^−2^ or higher, which minimizes the use of inactive components in the assembly. At the cell level, this has the effect of increasing the energy density and reducing the total cost (Lin et al., 2018; Chen et al., 2019; Molina et al., 2020). Although the performance of a given organic cathode will decrease when cycled at a higher loading, such a test allows for a preliminary evaluation of the material in a more realistic setting. To this end, there have been numerous reports of aqueous ZIBs functioning with loadings up to 30 mg cm^−2^ (Khayum et al., 2019; Huang et al., 2020; Zhang et al., 2020c; Patil et al., 2021b).

It is interesting to note that materials with modest specific capacities may be substantially more attractive if they are able to form thick electrode films and, therefore, have high areal capacities (and so higher energy densities). With this in mind, researchers can leverage molecular tuning with electrode film engineering to obtain the high areal capacities required for next-generation grid storage. A realistic target to strive toward is a capacity of 5 mAh cm^−2^ which can be sustained over a commercially relevant lifetime (i.e several thousands of cycles) at a practical charging rate (Ma et al., 2020).

When conducting high loading tests, the cycling performance of the anode should not be neglected. As the same amount of charge flows through the anode as the cathode, the active material loading dictates the amount of zinc being anodically stripped and plated. In typical academic experiments with low loadings, only a small fraction of the anode is reversibly cycled—in other words, the utilization ratio of the anode is small. Here, degradation of the anode can be masked by the large excess of zinc, which effectively acts as a reservoir (Lee et al., 2020). As the cathode loading is increased, the zinc utilization ratio grows and the reversibility of the anode becomes an increasingly limiting factor. At high loadings, device lifetime may be more reflective of anodic side-reactions or dendrite growth than of cathode stability. To address this, there are several strategies that have been developed to enhance the reversibility of zinc anodes, such as electrode coatings, zinc hosts, and electrolyte modification. These have been extensively reviewed elsewhere (Shin et al., 2020).

### Use of non-corrosive aqueous electrolytes in the molar range

A benefit of ZIBs over LIBs is their compatibility with aqueous electrolytes, which are advantageous due to the safety and high ionic conductivity of water. Moreover, as water costs very little (relative to organic solvents), the overall electrolyte system should be inexpensive based on solvent considerations alone. However, many batteries employ highly concentrated water-in-salt electrolytes (WiSE) (Wang et al., 2018; Song et al., 2021), which can widen the electrochemical window and improve cycling performance by greatly suppressing the activity of water. It is important to note that such systems can suffer from very high costs which can be tolerated in research settings but will present major barriers to commercialization beyond the laboratory (Chao and Qiao 2020). In this regard, the use of electrolytes in the molar range is a more promising route to take for materials targeting grid application.

Recently, a set of aqueous electrolytes based on molecular crowding have emerged as highly promising alternatives to WiSE systems. These electrolytes, whose solvents are a mixture of water and a molecular crowding agent, such as poly(ethylene glycol), function similarly to their WiSE counterparts. Briefly, the crowding agent interacts strongly with water, thereby greatly reducing the activity of free water molecules at the electrode/electrolyte interfaces (Xie et al., 2020). This endows molecular crowding electrolytes with the advantages of WiSE systems (reversibility, expanded voltage window) while avoiding their prohibitively high costs.

Another point to consider when employing aqueous electrolytes is the possibility for storage of ions beyond naked Zn^2+^. Recent reports have shown that Zn(H_2_O)_x_ and H^+^ can penetrate into organic cathodes and participate in the redox mechanism of the active material (Wan et al., 2018; Nam et al., 2020; Wang et al., 2020b; Yang et al., 2021). H^+^ storage is especially influential on the device’s electrochemical performance. For example, proton-storing materials typically display enhanced capacities due to the additional storage mode and very high rate capabilities owing to the fast kinetics of H^+^ coordination. This was recently exemplified by Tie and coworkers, whose proton-storing phenazine-based cathode maintained a capacity of 123 mAh g^−1^ at 20 A g^−1^ (one charge/discharge cycle in 44 s) (Tie et al., 2020).

However, H^+^ storage is accompanied by a local increase in pH at the electrode/electrolyte interface. This environmental change leads to the precipitation of insulating layered double hydroxides (LDH) onto the cathode surface. The LDH composition is Zn_4_(OH)_6_SO_4_·*x*H_2_O in ZnSO_4_, where it has best been characterized, but varies according to the electrolyte anion. The LDH flakes act as a pH buffer but their accumulation over extended cycling can lead to an increased electrode impedance and continuous electrolyte consumption (Zhang et al., 2020b). Furthermore, in discharged states, the LDH can break away from the cathode, thus eliminating the buffer. This leaves the interface susceptible to severe pH changes during H^+^ (un)coordination, which is detrimental to the long-term stability of the cell (Blanc et al., 2020). Notably, this behavior is masked at high cycling currents, and devices can be mistakenly deemed more stable than they are when cycled too fast. Cycling protocols involving rest periods in the discharged state can help identify H^+^ storage and associated LDH formation. The charge storage mechanism can also be characterized through cyclic voltammetry, *ex situ* measurements, and density functional theory.

Interestingly, some electrolyte classes can afford control over the mode of charge storage. By reducing the degree of free water and tuning the zinc solvation sheath, the storage of naked Zn^2+^ can be promoted while suppressing that of H^+^. This was recently achieved by Nazar and coworkers with a ZnCl_2_ WiSE system, as well as with a more sustainable molecular crowding electrolyte containing 70% poly(ethylene glycol) (Li et al., 2022).

Finally, it should be noted that other multivalent-ion batteries have suffered from corrosion of the current collectors and cell casing due to chloride-containing electrolytes (Yagi et al., 2013; Lipson et al., 2015). This may pose problems for researchers using ZnCl_2_ and can be alleviated by using less aggressive salts such as ZnSO_4_ and Zn(CF_3_SO_3_)_2_, which are widely employed in state-of-the-art electrolyte systems. For ZIBs, corrosion can not only be problematic for the non-active cell components but also for the metallic zinc anode itself. Here, corrosion can lead to passivation of the zinc surface as well as the evolution of hydrogen gas. The latter poses severe safety issues due to the possibility of dangerous pressure build-up in the sealed cells. The suppression of these detrimental side-reactions is an active research field in its own right, and electrolyte design strategies have been reviewed elsewhere (Huang et al., 2019; Liu et al., 2021; Bayaguud et al., 2022).

### Design and assessment of long-term stability

For grid applications, it is essential that batteries are able to withstand cycling over a long period of time. Organic molecules are typically sparingly soluble in water when oxidized but may experience dissolution in the aqueous electrolyte when in their Zn-coordinated reduced state, which results in capacity fading over repeated cycling. However, this can be mitigated by using compounds with high molecular weights, which are inherently less soluble than their small molecule counterparts. For example, Stoddart and coworkers showed that a triangular phenanthrenequinone-based macrocycle with a rigid geometry exhibited less dissolution and an improved structural stability compared to its phenanthrenequinone small molecule analog (Nam et al., 2020). Furthermore, cycling stability can be improved by tethering the small molecule to a polymeric backbone or integrating it into a covalent organic framework (COF) (Schon et al. 2017, 2019). In this way, the redox-active sites of the small molecule are still available, but the covalently linked macrostructure restricts the material’s solubility in the aqueous electrolyte. Such strategies have already been extended to ZIBs (Koshika et al., 2009; Khayum et al., 2019; Nam et al., 2020; Zhao et al., 2020). Another approach to reducing solubility in aqueous electrolytes is to design molecules with high symmetry and, therefore, low dipole moments, as was recently shown by Sun and coworkers (Lin et al., 2021).

A clear assessment of the cell’s cycling stability is important. The Coulombic efficiency (CE) serves as an important figure of merit for evaluating stability and is often shown in long-term cycling plots. The capacity retention of a metal-ion battery after some number of cycles can be approximated as:

$$\mathrm{Remaining Capacity}\;=\;CE^{\text{number of cycles}}$$

Therefore, even small differences in the CE can have a drastic impact on stability. Because of this, it is important to (i) report CE and (ii) use an appropriate axis to highlight minor changes in CE during cycling (Talaie et al., 2017). Finally, cycling stability tests should be conducted at relevant current densities to best reflect material degradation, dissolution, and LDH formation/detachment (if there is any) in a realistic setting.

#### Outlook

**Figure 3:**
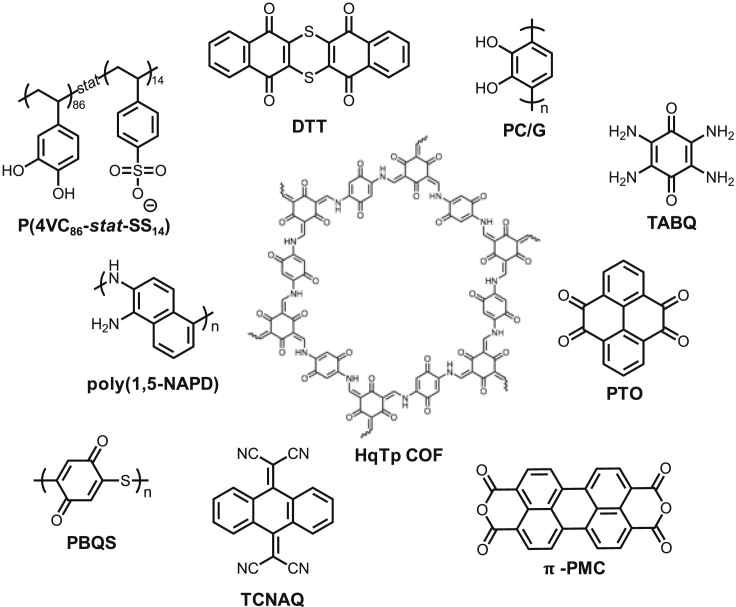
The molecular structures of cathode examples from Table 1

To illustrate the current state of organic ZIB cathode materials, we have tabulated several examples from the recent literature (Figure 3) and graded them based on how well they adhere to our design principles (Table 1). This not only provides a means of evaluating these different materials in terms of their application potential but it also highlights the challenges currently facing the field and, consequently, where future research may be directed.

**Table 1:** Evaluation of highlighted ZIB cathode materials

Cathode Material	Cost^a^	Simplicity, Scalability, Safety^b^	End-of-Life Scenarios^c^	Areal Loading^d^	Electrolyte^e^	Long-term stability^f^	Charge Storage Mechanism	Ref.
DTT	A	A	C	B	A	B	Zn^2+^ and H^+^	Wang et al., (2020b)
PC/G	C	B	C	C	B	A	Zn^2+^	Zhang et al., (2020d)
TABQ	A	A	C	C	A	B	H^+^	Lin et al., (2021)
P(4VC_86_-*stat*-SS_14_)	C	C	C	A	B	A	Zn^2+^	Patil et al., (2021a); Patil et al., (2021b)
HqTp COF	C	A	C	A	B	A	Zn^2+^	Khayum et al., (2019)
poly(1,5-NAPD)	A	A	C	B	A	B	Zn^2+^ and H^+^	Zhao et al., (2020)
PTO	A	A	C	B	A	C	Zn^2+^	Guo et al., (2018)
PBQS	B	B	C	C	B	B	Zn^2+^	Dawut et al., (2018)
TCNAQ	C	B	C	A	A	A	Zn^2+^	Wang et al., (2020a)
π-PMC	A	B	C	A	C	B	Zn^2+^	Zhang et al., (2020a)

A is the highest grade, while C is the lowest. An A requires the following criteria to be fulfilled

a The prototype cell does not make use of graphene or Nafion membranes and the synthesis does not make use of highly expensive reagents (ie. above $100/g on the lab scale).

b The synthesis does not require inert conditions or temperatures above 100°C, takes 3 steps or fewer, and does not require highly dangerous reagents.

c End-of-life scenarios such as recycling or biodegradation are considered for the material.

d An active material loading of at least 10 mg cm^−2^ has been tested.

e The electrolyte is no more than 2 M in concentration and is not based on ZnCl_2_.

f The prototype cell retains 80% of its capacity for at least 1000 cycles and primarily stores Zn^2+^.

We note that it was difficult to accurately assess and compare the stabilities of the different examples as the current densities used for the cycling tests vary widely between reports. Stabilities should ideally be evaluated at realistic rates so as to better gauge the long-term performance of devices and to observe any deleterious effects arising from the storage of H^+^ (see Electrolyte section).

It can be seen in Table 1 that recent organic ZIB cathodes are fulfilling many of the criteria desired of a sustainable grid material. In general, great progress has been made toward increasing the electrochemical performance of organic electrodes, which is partly due to the successful translation of strategies employed in LIB research. However, further research should be targeted at increasing the intrinsic stability and energy density of materials through molecular tuning, which is an inherently low-cost approach to obtaining high-performance devices with long lifetimes. For instance, there are many strategies, such as polymerization, salification, and hybridization with insoluble substrates (i.e carbon materials), that can be used to improve cycling stability. Likewise, the output voltage of organic batteries can be modified through a handful of well-known approaches. For example, adjusting the LUMO level by incorporating electron-withdrawing groups (i.e -CF_3_, -CN, -F, -Cl, -Br, and -SO_3_Na) or electron-donating groups (i.e -NH_2_, -CH_3_, -OCH_3_, -OLi, and -ONa) is a facile way to increase or decrease the working potential. As well, the relative positions of active groups (i.e ortho, para, or meta) are significant, as they change the discharge potential due to different Coulombic interactions. Finally, rate performance can be improved by designing molecules with extended π-conjugated structures and additional conductive units (Lu et al., 2018).

In terms of synthetic complexity, the methods used to produce current organic electrodes range widely from controlled polymerizations to solid-state (i.e mechanochemical) syntheses. Although the synthetic route of a material may appear to be a trivial concern, it has a significant impact upon the material’s applicability, where reaction concentration, yield, complexity, material cost, and number of steps must be considered. This is especially true for a technology that is conceived to operate on the very large scale of grid storage. Therefore, developing sustainable and scalable routes to novel or established electrode materials represent a valuable contribution to the field. In this respect, we view bio-inspired chemistry and mechanochemistry as particularly attractive methodologies due to their use of aqueous and solvent-free conditions, respectively. Similarly, synthesizing electrode materials through flow chemistry can serve as a useful proof of concept for reaction scalability, as many industrial polymerizations are performed through continuous (rather than batch) processes (Zaquen et al., 2020).

As with other organic batteries, a key challenge is bridging the gap between laboratory-scale cells and commercially relevant energy storage. To do so, the successful cycling of an electrode with a high proportion of active material and a high mass loading can be seen as an important milestone in the development of a grid storage material. Currently, the low electrical conductivity of organic materials, which needs to be compensated with additives in the cathode film, continues to be an issue and requires further work. On the other hand, the testing of organic cathodes at high mass loadings (i.e 10 mg cm^−2^ or higher) is gaining traction. Such tests provide a means of evaluating the suitability of an organic material for use in a realistic grid battery. The engineering of thick electrodes is a viable route to making significant gains in areal capacity, which is a key metric for evaluating the performance of grid-based battery materials.

Another major conclusion is the urgent need to design electrode materials for biodegradation from conception. To the best of our knowledge, there are no examples of organic ZIB electrodes specifically designed to have a positive end-of-life scenario. When considering the high potential of organic materials for molecular modification, this is clearly a wasted opportunity. For example, synthesizing polymer electrode materials with hydrolyzable backbones (e.g polyesters) and highly amorphous morphologies may be a viable path toward engineering biodegradation (Tokiwa et al., 2009; Hatti-Kaul et al., 2020). Therefore, the design of electrode materials with end-of-life considerations is a key point of emphasis for future ZIB research.

In summary, aqueous-organic ZIBs offer a promising solution to the anticipated surge in demand for grid energy storage. For this technology to fulfill its potential, cathode materials must be developed with an emphasis on practicality and sustainability. In this respect, further work targeted at lowering cost, developing sustainable and scalable syntheses, and engineering positive end-of-life scenarios are of paramount importance. Nevertheless, recent reports of highly stable organic electrodes at commercially relevant mass loadings offer optimism for the future application of aqueous zinc-organic batteries. As with other energy storage technologies, the adoption of these batteries will require the sustained and concerted research and development efforts of chemists, process engineers, materials scientists, and policy makers to reach fruition.

## Supplemental information

Table S1 Summary of cathode materials

## References

[bib1] An S.Y., McAllister B.T., Grignon E., Evariste S., Seferos D.S. (2022). Increasing ionic conductivity in polymer electrodes using oxanorbornene. EcoMat.

[bib2] An S.Y., Schon T.B., Seferos D.S. (2020). Stable, dual redox unit organic electrodes. ACS Omega.

[bib3] Bayaguud A., Fu Y., Zhu C. (2022). Interfacial parasitic reactions of zinc anodes in zinc ion batteries: underestimated corrosion and hydrogen evolution reactions and their suppression strategies. J. Energy Chem..

[bib4] Blanc L.E., Kundu D., Nazar L.F. (2020). Scientific challenges for the implementation of Zn-ion batteries. Joule.

[bib5] Canepa P., Sai Gautam G., Hannah D.C., Malik R., Liu M., Gallagher K.G., Persson K.A., Ceder G. (2017). Odyssey of multivalent cathode materials: open questions and future challenges. Chem. Rev..

[bib6] Chao D., Zhou W., Ye C., Zhang Q., Chen Y., Gu L., Davey K., Qiao S.Z. (2019). An electrolytic Zn–MnO2 battery for high-voltage and scalable energy storage. Angew. Chem. Int. Edition.

[bib7] Chao D., Qiao S.-Z. (2020). Toward high-voltage aqueous batteries: super- or low-concentrated electrolyte?. Joule.

[bib8] Chen S., Niu C., Lee H., Li Q., Yu L., Xu W., Zhang J.G., Dufek E.J., Whittingham M.S., Meng S. (2019). Critical parameters for evaluating coin cells and pouch cells of rechargeable Li-metal batteries. Joule.

[bib9] Cui J., Guo Z., Yi J., Liu X., Wu K., Liang P., Li Q., Liu Y., Wang Y., Xia Y., Zhang J. (2020). Organic cathode materials for rechargeable zinc batteries: mechanisms, challenges, and perspectives. ChemSusChem.

[bib10] Dawut G., Lu Y., Miao L., Chen J. (2018). High-performance rechargeable aqueous Zn-ion batteries with a poly(benzoquinonyl sulfide) cathode. Inorg. Chem. Front..

[bib11] Dunn B., Kamath H., Tarascon J.-M. (2011). Electrical energy storage for the grid: a battery of choices. Science.

[bib12] Guo Z., Ma Y., Dong X., Huang J., Wang Y., Xia Y. (2018). An environmentally friendly and flexible aqueous zinc battery using an organic cathode. Angew. Chem..

[bib13] Gür T.M. (2018). Review of electrical energy storage technologies, materials and systems: challenges and prospects for large-scale grid storage. Energy Environ. Sci..

[bib14] Harper G., Sommerville R., Kendrick E., Driscoll L., Slater P., Stolkin R., Walton A., Christensen P., Heidrich O., Lambert S. (2019). Recycling lithium-ion batteries from electric vehicles. Nature.

[bib15] Hatti-Kaul R., Nilsson L.J., Zhang B., Rehnberg N., Lundmark S. (2020). Designing biobased recyclable polymers for plastics. Trends Biotechnol..

[bib16] Huang C., Zhao X., Xu Y., Zhang Y., Yang Y., Hu A., Tang Q., Song X., Jiang C., Chen X. (2020). Sewable and cuttable flexible zinc-ion hybrid supercapacitor using a polydopamine/carbon cloth-based cathode. ACS Sustainable Chem. Eng..

[bib17] Huang S., Zhu J., Tian J., Niu Z. (2019). Recent progress in the electrolytes of aqueous zinc-ion batteries. Chem. – A Eur. J..

[bib18] Iturrondobeitia M., Akizu-Gardoki O., Amondarain O., Minguez R., Lizundia E. (2022). Environmental impacts of aqueous zinc ion batteries based on life cycle assessment. Adv. Sustainable Syst..

[bib19] Khayum M.A., Ghosh M., Vijayakumar V., Halder A., Nurhuda M., Kumar S., Addicoat M., Kurungot S., Banerjee R. (2019). Zinc ion interactions in a two-dimensional covalent organic framework based aqueous zinc ion battery. Chem. Sci..

[bib20] Kolstad J.J., Vink E.T.H., De Wilde B., Debeer L. (2012). Assessment of anaerobic degradation of Ingeo^TM^ polylactides under accelerated landfill conditions. Polym. Degrad. Stab..

[bib21] Koshika K., Sano N., Oyaizu K., Nishide H. (2009). An aqueous, electrolyte-type, rechargeable device utilizing a hydrophilic radical polymer-cathode. Macromolecular Chem. Phys..

[bib22] Kundu D., Adams B.D., Duffort V., Vajargah S.H., Nazar L.F. (2016). A high-capacity and long-life aqueous rechargeable zinc battery using a metal oxide intercalation cathode. Nat. Energy.

[bib23] Kundu D., Oberholzer P., Glaros C., Bouzid A., Tervoort E., Pasquarello A., Niederberger M. (2018). Organic cathode for aqueous Zn-ion batteries: taming a unique phase evolution toward stable electrochemical cycling. Chem. Mater..

[bib24] Larcher D., Tarascon J.-M. (2015). Towards greener and more sustainable batteries for electrical energy storage. Nat. Chem..

[bib25] Lee S., Kang I., Kim J., Kim S., Kang K., Hong J. (2020). Real-time visualization of Zn metal plating/stripping in aqueous batteries with high areal capacities. J. Power Sourc..

[bib26] Li C., Kingsbury R., Zhou L., Shyamsunder A., Persson K.A., Nazar L.F. (2022). Tuning the solvation structure in aqueous zinc batteries to maximize Zn-ion intercalation and optimize dendrite-free zinc plating. ACS Energy Lett..

[bib27] Li C.-J., Trost B.M. (2008). Green chemistry for chemical synthesis. Proc. Natl. Acad. Sci..

[bib28] Liang Y., Jing Y., Gheytani S., Lee K.-Y., Liu P., Facchetti A., Yao Y. (2017). Universal quinone electrodes for long cycle life aqueous rechargeable batteries. Nat. Mater..

[bib29] Lin Z., Liu T., Ai X., Liang C. (2018). Aligning academia and industry for unified battery performance metrics. Nat. Commun..

[bib30] Lin Z., Shi H.-Y., Lin L., Yang X., Wu W., Sun X. (2021). A high capacity small molecule quinone cathode for rechargeable aqueous zinc-organic batteries. Nat. Commun..

[bib31] Lipson A.L., Proffit D.L., Pan B., Fister T.T., Liao C., Burrell A.K., Vaughey J.T., Ingram B.J. (2015). Current collector corrosion in Ca-ion batteries. J. Electrochem. Soc..

[bib32] Liu C., Xie X., Lu B., Zhou J., Liang S. (2021). Electrolyte strategies toward better zinc-ion batteries. ACS Energy Lett..

[bib33] Lu Y., Zhang Q., Li L., Niu Z., Chen J. (2018). Design strategies toward enhancing the performance of organic electrode materials in metal-ion batteries. Chem.

[bib34] Ma L., Chen S., Long C., Li X., Zhao Y., Liu Z., Huang Z., Dong B., Zapien J.A., Zhi C. (2019). Achieving high-voltage and high-capacity aqueous rechargeable zinc ion battery by incorporating two-species redox reaction. Adv. Energy Mater..

[bib35] Ma L., Schroeder M.A., Borodin O., Pollard T.P., Ding M.S., Wang C., Xu K. (2020). Realizing high zinc reversibility in rechargeable batteries. Nat. Energy.

[bib36] McAllister B.T., Grignon E., Schon T.B., An S.Y., Yim C.-H., Abu-Lebdeh Y., Seferos D.S. (2021). High-rate activation of organic superlithiation anodes. ACS Appl. Energy Mater..

[bib37] Molina A., Patil N., Ventosa E., Liras M., Palma J., Marcilla R. (2020). Electrode engineering of redox-active conjugated microporous polymers for ultra-high areal capacity organic batteries. ACS Energy Lett..

[bib38] Nam K.W., Kim H., Beldjoudi Y., Kwon T.W., Kim D.J., Stoddart J.F. (2020). Redox-active phenanthrenequinone triangles in aqueous rechargeable zinc batteries. J. Am. Chem. Soc..

[bib39] Nguyen T.P., Easley A.D., Kang N., Khan S., Lim S.M., Rezenom Y.H., Wang S., Tran D.K., Fan J., Letteri R.A. (2021). Polypeptide organic radical batteries. Nature.

[bib40] Pan C., Zhang R., Nuzzo R.G., Gewirth A.A. (2018). ZnNixMnxCo2–2xO4 Spinel as a High-Voltage and High-Capacity Cathode Material for Nonaqueous Zn-Ion Batteries. Adv. Energy Mater..

[bib41] Pan H., Shao Y., Yan P., Cheng Y., Han K.S., Nie Z., Wang C., Yang J., Li X., Bhattacharya P. (2016). Reversible aqueous zinc/manganese oxide energy storage from conversion reactions. Nat. Energy.

[bib42] Patil N., de la Cruz C., Ciurduc D., Mavrandonakis A., Palma J., Marcilla R. (2021). An ultrahigh performance zinc-organic battery using poly(catechol) cathode in Zn(TFSI)2-Based concentrated aqueous electrolytes. Adv. Energy Mater..

[bib43] Patil N., Palma J., Marcilla R. (2021). Macromolecular engineering of poly(catechol) cathodes towards high-performance aqueous zinc-polymer batteries. Polymers.

[bib44] Poizot P., Gaubicher J., Renault S., Dubois L., Liang Y., Yao Y. (2020). Opportunities and challenges for organic electrodes in electrochemical energy storage. Chem. Rev..

[bib45] Powell J.T., Townsend T.G., Zimmerman J.B. (2016). Estimates of solid waste disposal rates and reduction targets for landfill gas emissions. Nat. Clim. Change.

[bib46] Schon T.B., An S.Y., Tilley A.J., Seferos D.S. (2019). Unusual capacity increases with cycling for ladder-type microporous polymers. ACS Appl. Mater. Inter..

[bib47] Schon T.B., Tilley A.J., Bridges C.R., Miltenburg M.B., Seferos D.S. (2016). Bio-derived polymers for sustainable lithium-ion batteries. Adv. Funct. Mater..

[bib48] Schon T.B., Tilley A.J., Kynaston E.L., Seferos D.S. (2017). Three-Dimensional arylene diimide frameworks for highly stable lithium ion batteries. ACS Appl. Mater. Inter..

[bib49] Shin J., Lee J., Park Y., Choi J.W. (2020). Aqueous zinc ion batteries: focus on zinc metal anodes. Chem. Sci..

[bib50] Song X., He H., Aboonasr Shiraz M.H., Zhu H., Khosrozadeh A., Liu J. (2021). Enhanced reversibility and electrochemical window of Zn-ion batteries with an acetonitrile/water-in-salt electrolyte. Chem. Commun..

[bib51] Talaie E., Bonnick P., Sun X., Pang Q., Liang X., Nazar L.F. (2017). Methods and protocols for electrochemical energy storage materials research. Chem. Mater..

[bib52] Tie Z., Liu L., Deng S., Zhao D., Niu Z. (2020). Proton insertion chemistry of a zinc–organic battery. Angew. Chem. Int. Edition.

[bib53] Tokiwa Y., Calabia B.P., Ugwu C.U., Aiba S. (2009). Biodegradability of plastics. Int. J. Mol. Sci..

[bib54] Trócoli R., La Mantia F. (2015). An aqueous zinc-ion battery based on copper hexacyanoferrate. ChemSusChem.

[bib55] Wan F., Zhang L., Wang X., Bi S., Niu Z., Chen J. (2018). An aqueous rechargeable zinc-organic battery with hybrid mechanism. Adv. Funct. Mater..

[bib56] Wang F., Borodin O., Gao T., Fan X., Sun W., Han F., Faraone A., Dura J.A., Xu K., Wang C. (2018). Highly reversible zinc metal anode for aqueous batteries. Nat. Mater..

[bib57] Wang Q., Xu X., Yang G., Liu Y., Yao X. (2020). An organic cathode with tailored working potential for aqueous Zn-ion batteries. Chem. Commun..

[bib58] Wang Y., Wang C., Ni Z., Gu Y., Wang B., Guo Z., Wang Z., Bin D., Ma J. (2020). Binding zinc ions by carboxyl groups from adjacent molecules toward long-life aqueous zinc–organic batteries. Adv. Mater..

[bib59] Xie J., Liang Z., Lu Y.-C. (2020). Molecular crowding electrolytes for high-voltage aqueous batteries. Nat. Mater..

[bib60] Xu C., Li B., Du H., Kang F. (2012). Energetic zinc ion chemistry: the rechargeable zinc ion battery. Angew. Chem. Int. Edition.

[bib61] Yagi S., Tanaka A., Ichikawa Y., Ichitsubo T., Matsubara E. (2013). Electrochemical stability of magnesium battery current collectors in a grignard reagent-based electrolyte. J. Electrochem. Soc..

[bib62] Yan M., He P., Chen Y., Wang S., Wei Q., Zhao K., Xu X., An Q., Shuang Y., Shao Y. (2018). Water-lubricated intercalation in V2O5·nH2O for high-capacity and high-rate aqueous rechargeable zinc batteries. Adv. Mater..

[bib63] Yang B., Ma Y., Bin D., Lu H., Xia Y. (2021). Ultralong-life cathode for aqueous zinc-organic batteries via pouring 9,10-phenanthraquinone into active carbon. ACS Appl. Mater. Inter..

[bib64] Zaquen N., Rubens M., Corrigan N., Xu J., Zetterlund P.B., Boyer C., Junkers T. (2020). Polymer synthesis in continuous flow reactors. Prog. Polym. Sci..

[bib65] Zhang H., Fang Y., Yang F., Liu X., Lu X. (2020). Aromatic organic molecular crystal with enhanced π–π stacking interaction for ultrafast Zn-ion storage. Energy Environ. Sci..

[bib66] Zhang N., Cheng F., Liu Y., Zhao Q., Lei K., Chen C., Liu X., Chen J. (2016). Cation-deficient spinel ZnMn2O4 cathode in Zn(CF3SO3)2 electrolyte for rechargeable aqueous Zn-ion battery. J. Am. Chem. Soc..

[bib67] Zhang N., Cheng F., Liu J., Wang L., Long X., Liu X., Li F., Chen J. (2017). Rechargeable aqueous zinc-manganese dioxide batteries with high energy and power densities. Nat. Commun..

[bib68] Zhang N., Dong Y., Jia M., Bian X., Wang Y., Qiu M., Xu J., Liu Y., Jiao L., Cheng F. (2018). Rechargeable aqueous Zn–V2O5 battery with high energy density and long cycle life. ACS Energy Lett..

[bib69] Zhang N., Chen X., Yu M., Niu Z., Cheng F., Chen J. (2020). Materials chemistry for rechargeable zinc-ion batteries. Chem. Soc. Rev..

[bib70] Zhang Q., Ma Y., Lu Y., Li L., Wan F., Zhang K., Chen J. (2020). Modulating electrolyte structure for ultralow temperature aqueous zinc batteries. Nat. Commun..

[bib71] Zhang S., Zhao W., Li H., Xu Q. (2020). Cross-conjugated polycatechol organic cathode for aqueous zinc-ion storage. ChemSusChem.

[bib72] Zhao Q., Huang W., Luo Z., Liu L., Lu Y., Li Y., Li L., Hu J., Ma H., Chen J. (2018). High-capacity aqueous zinc batteries using sustainable quinone electrodes. Sci. Adv..

[bib73] Zhao Y., Wang Y., Zhao Z., Zhao J., Xin T., Wang N., Liu J. (2020). Achieving high capacity and long life of aqueous rechargeable zinc battery by using nanoporous-carbon-supported poly(1,5-naphthalenediamine) nanorods as cathode. Energy Storage Mater..

[bib74] Zhou J., Shan L., Wu Z., Guo X., Fang G., Liang S. (2018). Investigation of V2O5 as a low-cost rechargeable aqueous zinc ion battery cathode. Chem. Commun..

