# Supplementary material for: Aqueous zinc batteries: Design principles toward organic cathodes for grid applications
Source: iScience. 2022 Apr 4;25(5):104204. doi: 10.1016/j.isci.2022.104204 (PMC9046109; doi:10.1016/j.isci.2022.104204)
Supplement: Table S1 — Summary of cathode materials [file mmc1.pdf]

iScience, Volume *25*

Supplemental information

Aqueous zinc batteries: Design principles toward organic cathodes for grid applications

Eloi Grignon, Alicia M. Battaglia, Tyler B. Schon, and Dwight S. Seferos

Supplemental Information

Table S1. Summary of electrochemical properties of cathode materials for ZIBs from Table 1.

Active Material	C_{Theor} (mAh g^{-1})	Electrode Composition	Electrolyte	Capacity (mAh g^{-1}), current	Average Discharge Potential (V) vs. Zn/Zn^{2+}	Cycling Stability (retention; cycles; current)	Ref.
**DTT**	285	60:30:10 (DTT: KB: PTFE)	2M $ZnSO_4$	210.9, 50 mA g^{-1}	0.8	84%<br>23000<br>2 A g^{-1}	Wang et al. 2020b
**PC/G**	500	60:120 (PC: GO) *hydrogel (no current collector)	3M $ZnSO_4$	355, 0.1 C<br>171, 10 C	1.25	74%<br>3000<br>2 C	Zhang et al. 2020d
**TABQ**	638	5:4:1 (TABQ: KB: PVDF)	1M $ZnSO_4$	303, 0.1 A g^{-1}<br>213, 5 A g^{-1}	1.0	n a<br>1000<br>5 A g^{-1}	Lin et al. 2021
**P(4VC$_{86}$-*stat*-SS$_{14}$)**	344	60:40 (P(4VC$_{86}$-*stat*-SS$_{14}$): MWCNTs) *buckypaper electrode	4M $Zn(TFSI)_2$	325- 279, <10 C	1.2	83%<br>48,000<br>30C	Patil et al. 2021a; Patil et al. 2021b
**HqTp COF**	442	3:1 (HqTp COF: CNF) + 5wt% Nafion solution	3M $ZnSO_4$	276, 125 mA g^{-1}	0.87	95%<br>1000<br>3750 mA g^{-1}	Khayum et al. 2019
**poly(1,5-NAPD)**	288	8:1:1 (AC powder: CB: PVDF) *electro-polymerized	2M $ZnSO_4$	315, 0.19 A g^{-1}	0.9	91%<br>10000<br>10 A g^{-1}	Zhao et al. 2020
**PTO**	409	60:30:10 (PTO: KB: PTFE)	2M $ZnSO_4$	336, 0.04 A g^{-1}	0.8	70%<br>1000<br>3 A g^{-1}	Guo et al. 2018
**PBQS**	388	60:30:10 (PBQS: CB: PVDF)	3M $Zn(OTf)_2$	203, 0.1 C	1	86%<br>50<br>0.2 C	Dawut et al. 2018
**TCNAQ**	176	60:30:10 (TCNAQ: super-P: PTFE)	2M $ZnSO_4$	169, 20 mA g^{-1}	1.1	81%<br>1000<br>500 mA g^{-1}	Wang et al. 2020a
**ππ-PMC**	136	80:10:10 (π-PMC: CB: PVDF)	2M $ZnCl_2$	123, 0.2 A g^{-1}	0.5	63%<br>500<br>32 A g^{-1}	Zhang et al. 2020a

n a = not available. Theoretical capacity (C_{Theo}) = (26801 x n)/ MW; n = number of electrons involved in the redox process(es), MW= molecular weight of the active material. Capacity retention (%) = ($C_{s,\ max\ or\ first\ cycle}$ / $C_{s,\ last\ cycle}$) x 100.
Zn- salts: zinc sulfate ($ZnSO_4$), zinc bis(trifluoromethanesulfonyl)imide ($Zn(TFSI)_2$), zinc trifluoromethanesulfonate ($Zn(OTf)_2$), zinc chloride ($ZnCl_2$)
Conductive additives: Ketjen black (KB), graphene oxide (GO), multi-walled carbon nanotubes (MWCNTs), carbon nanofibres (CNF), carbon black (CB), acetylene black (AB), activated carbon (AC)
Binders: poly(tetrafluoroethylene) (PTFE), poly(vinylidenedifluoride) (PVDF)

References

Dawut, G., Lu, Y., Miao, L. and Chen, J. 2018. High-performance rechargeable aqueous Zn-ion batteries with a poly(benzoquinonyl sulfide) cathode. *Inorganic Chemistry Frontiers* 5(6), pp. 1391–1396. doi: 10.1039/C8QI00197A.
Guo, Z., Ma, Y., Dong, X., Huang, J., Wang, Y. and Xia, Y. 2018. An Environmentally Friendly and Flexible Aqueous Zinc Battery Using an Organic Cathode. *Angewandte Chemie* 130(36), pp. 11911–11915. doi: 10.1002/ange.201807121.
Khayum, A.M. et al. 2019. Zinc ion interactions in a two-dimensional covalent organic framework based aqueous zinc ion battery. *Chemical Science* 10(38), pp. 8889–8894. doi: 10.1039/c9sc03052b.
Lin, Z., Shi, H.-Y., Lin, L., Yang, X., Wu, W. and Sun, X. 2021. A high capacity small molecule quinone cathode for rechargeable aqueous zinc-organic batteries. *Nature Communications* 12(1), p. 4424. doi: 10.1038/s41467-021-24701-9.
Patil, N., de la Cruz, C., Ciurduc, D., Mavrandonakis, A., Palma, J. and Marcilla, R. 2021a. An Ultrahigh Performance Zinc-Organic Battery using Poly(catechol) Cathode in Zn(TFSI)2-Based Concentrated Aqueous Electrolytes. *Advanced Energy Materials* 11(26), p. 2100939. doi: 10.1002/aenm.202100939.
Patil, N., Palma, J. and Marcilla, R. 2021b. Macromolecular Engineering of Poly(catechol) Cathodes towards High-Performance Aqueous Zinc-Polymer Batteries. *Polymers* 13(11), p. 1673. doi: 10.3390/polym13111673.
Wang, Y. et al. 2020b. Binding Zinc Ions by Carboxyl Groups from Adjacent Molecules toward Long-Life Aqueous Zinc–Organic Batteries. *Advanced Materials* 32(16), p. 2000338. doi: 10.1002/adma.202000338.
Wang, Q., Xu, X., Yang, G., Liu, Y. and Yao, X. 2020a. An organic cathode with tailored working potential for aqueous Zn-ion batteries. *Chemical Communications* 56(79), pp. 11859–11862. doi: 10.1039/D0CC05344A.
Zhang, S., Zhao, W., Li, H. and Xu, Q. 2020d. Cross-Conjugated Polycatechol Organic Cathode for Aqueous Zinc-Ion Storage. *ChemSusChem* 13(1), pp. 188–195. doi: 10.1002/cssc.201902697.
Zhang, H., Fang, Y., Yang, F., Liu, X. and Lu, X. 2020a. Aromatic organic molecular crystal with enhanced π–π stacking interaction for ultrafast Zn-ion storage. *Energy & Environmental Science* 13(8), pp. 2515–2523. doi: 10.1039/D0EE01723J.
Zhao, Y., Wang, Y., Zhao, Z., Zhao, J., Xin, T., Wang, N. and Liu, J. 2020. Achieving high capacity and long life of aqueous rechargeable zinc battery by using nanoporous-carbon-supported poly(1,5-naphthalenediamine) nanorods as cathode. *Energy Storage Materials* 28, pp. 64–72. doi: 10.1016/j.ensm.2020.03.001.
